# The production of information in the attention economy

**DOI:** 10.1038/srep09452

**Published:** 2015-05-19

**Authors:** Giovanni Luca Ciampaglia, Alessandro Flammini, Filippo Menczer

**Affiliations:** 1 Center for Complex Networks and Systems Research, School of Informatics and Computing, Indiana University, Bloomington, IN 47408, USA

## Abstract

Online traces of human activity offer novel opportunities to study the dynamics of complex knowledge exchange networks, in particular how emergent patterns of collective attention determine what new information is generated and consumed. Can we measure the relationship between demand and supply for new information about a topic? We propose a normalization method to compare attention bursts statistics across topics with heterogeneous distribution of attention. Through analysis of a massive dataset on traffic to Wikipedia, we find that the production of new knowledge is associated to significant shifts of collective attention, which we take as proxy for its demand. This is consistent with a scenario in which allocation of attention toward a topic stimulates the demand for information about it, and in turn the supply of further novel information. However, attention spikes only for a limited time span, during which new content has higher chances of receiving traffic, compared to content created later or earlier on. Our attempt to quantify demand and supply of information, and our finding about their temporal ordering, may lead to the development of the fundamental laws of the attention economy, and to a better understanding of social exchange of knowledge information networks.

Massive logs on online human activity create new possibilities to study complex socio-economic phenomena^1^,^2^,^3^. Among these, the dynamics of knowledge exchange networks, and in particular the emergent interactions between producers and consumers of information^4^, have not been explored like the flows of material goods. Yet they have a critical impact on our opinions, decisions, and lives^5^.

An overwhelming amount of information stimuli compete for our cognitive resources, giving rise to the *economy of attention*^6^, first theorized by Simon^7^. At the aggregate level, this phenomenon is often referred to as *collective attention*. Work on collective attention has mainly focused on the *consumption* of information^8^,^9^,^10^. Characteristic signatures of information consumption have been shown to correlate with real-world events, such as the spread of influenza^11^, financial stock returns^2^, scientific performance^12^, and box office results^13^.

The *production* side of the equation — whether and how the creation of information is driven by demand — has been explored to a limited extent in the literature, owing in part to the challenges in quantifying information demand. Imitation of popular content^14^, for instance, is the simplest form of supply matching demand for information. However, while examples of imitation of online contents abound^15^, they do not point to a quantitative relationship between the demand for and production of information. In looking at the role of attention as a possible driver for the generation of novel content, Huberman *et al*. found a positive correlation between the productivity of YouTube contributors and the number of views of their previous videos^16^. This confirms that prestige is a powerful motivation for creation of knowledge^17^.

Here we tackle the measurement of demand and supply of information goods and their relative ordering in time. Looking at attention toward a specific piece of information, no link between traffic bursts and the number of edits to a Wikipedia article has been found so far^18^. We focus on the *creation* of Wikipedia articles as a better proxy for the production of information, and on visits to topically related articles as a proxy for its demand. Analysis of Wikipedia traffic data thus allows us to study how the generation of new knowledge about a topic precedes or follows its demand.

More specifically, we are interested in how attention toward topics changes around the time that new knowledge about them is created. Moreover, we want to do so by comparing a broad range of topics. Sudden changes of attention, or “bursts”, have been traditionally studied using the logarithmic derivative Δ*N_t_*/*N_t_*, where *N_t_* is the number of visits or links accrued by a topic (e.g. a Wikipedia page, a YouTube video, etc.) during a fixed sampling interval *t* and the numerator is customarily defined as Δ*N_t_* = *N_t_*_+1_ − *N_t_*^18^,^19^,^20^. However, the distribution of Δ*N*/*N* is known to be broad, with a heavy-tail decay that follows a power-law distribution^19^. This lack of a characteristic scale thus makes it difficult to use Δ*N*/*N* for comparing diverse topics. Here we propose to use a different measure of traffic change based on a simple normalization of the traffic, in a way that takes into account this and other confounding factors, such as traffic seasonality and circadian rhythms of activity^21^,^22^.

Wikipedia is currently the fifth most visited Internet website^23^, and includes 30 million articles in 287 languages. The English version alone consists of roughly 4.4 million articles and is consulted, on average, by about 300 million people every day. Each entry, or article, of Wikipedia corresponds to a separate web page. Wikipedia can thus be regarded as a large information network, where one can identify broad macroscopic topics. By way of example, Fig. 1 depicts the traffic to two high-profile articles and to their neighbors. The two articles are selected from the 2012 Google Zeitgeist^24^. We define a topic as such a page, together with all of its neighbors — articles linked by it or linking to it, subsequently to its creation (see Methods). The networks formed by the two topics are shown in Fig. 1(b, d).

The volume of traffic to a page or a topic is measured by daily browser requests for the corresponding pages. Weekly fluctuations are evident in the traffic patterns shown in Fig. 1(a, c). It is also possible to observe synchronous bursts of activity, corresponding to increased attention toward the topic. For the Olympics topic, such increase of attention takes the form of an anticipatory buildup, leading to two peaks around the opening and closing ceremonies, followed by a relaxation at a lower baseline. For Hurricane Sandy a sudden spike occurs at the time of creation of the main article, due to the demand of information about the effects of the hurricane.

Phenomena like these have been already observed in a wide range of information-rich environments^1^,^14^,^19^,^20^,^25^. During the period of increased attention we see a previously unobserved phenomenon, namely that new articles about the Olympic Games are created at a higher frequency. A weaker pattern is observed for Hurricane Sandy too. To quantify the temporal relation between demand and production of information about a topic, we performed a systematic study over a large sample of articles. An increase of attention toward the topic of an article is revealed by an increase in requests for pages in that topic compared to other topics.

Let us consider a newly created article. A burst of attention for pages related to it occurring before its creation is consistent with a model in which demand drives the supply of information. Conversely, a burst that follows its creation suggests that demand follows supply. On the other hand, if traffic bursts concomitant with the creation of new articles are no different than those observed at any other time, then we shall conclude that production and consumption of information are two unrelated processes.

As the focus of public attention is constantly shifting, we also explore how long is the timespan during which demand and supply for new information are effectively associated to each other. In other words, is there an ideal period during which newly created information will have better chances of receiving more traffic relative to its baseline? We address this question by measuring the time lag between the most recent peak of traffic toward the pages in the topic of a new article and its time of creation. We relate this lag to the traffic received by the new article.

## Results

### Statistics of attention bursts

Our analysis is focused on the year 2012. We collected the neighbors of 93,491 pages created during that year. For each created page we considered the two weeks before and after its creation, and measured the volume of traffic to its topic in each week. We characterize the typical traffic to the topic in the week after and before with the median traffic to neighbors *V*^(*a*)^ and *V*^(*b*)^, respectively. Let us define infra-week traffic volume change Δ*V* = *V*^(*a*)^ − *V*^(*b*)^, total volume *V* = *V*^(*a*)^ + *V*^(*b*)^, and relative volume change Δ*V*/*V*.

For comparison purposes, we collected the neighbors of a roughly equally-sized sample of pre-existing articles (created before 2012) and analogously computed their relative infra-week changes in traffic volume over random two-week windows in 2012. Articles in the baseline sample are older and therefore tend to have more neighbors, as shown in Fig. 2(a). This and other temporal effects are discounted by considering the relative change in volume Δ*V*/*V* (see Methods).

We observe that the volume change |Δ*V*| scales sublinearly with the total volume *V*, as illustrated in Fig. 2(b). Consequently |Δ*V*|/*V* goes to zero as *V* increases. While the distributions of Δ*V*/*V* are sharply peaked around zero in both samples (Fig. 2(c)), they are different: a non-parametric Kolmogorov-Smirnov test rejects the null hypothesis that the two samples of relative traffic change are drawn from the same distribution (*D* = 0.034, *p* < 0.001); an Anderson-Darling test, which gives less weight to the median values of the distribution in favor of the tails, yields similar results. One way to quantify and interpret the difference between the two distributions is to compute the ratio of odds that a given change in traffic volume is observed when a page is created versus when a page has existed for a specific amount of time. Fig. 2(d) plots the log odds as a function of Δ*V*/*V*. For example Δ*V*/*V* = −0.5 is over two orders of magnitude more likely to be observed in a new page compared to a page of generic but fixed age. As shown in the figure, this effect holds even when we consider only neighborhoods with a high volume of traffic, which may be indicative of more developed, and hence more popular topics. In summary, while we find both instances in which bursts in demand precede (Δ*V*/*V* < 0) and follow (Δ*V*/*V* > 0) the generation of new knowledge, comparison with the baseline yields a significant shift toward the former case, suggesting that consumption anticipates the production of information more often than the converse.

### Top attention bursts

Which kinds of articles precede or follow demand for information? In Table 1 we list a few articles with the largest positive and negative bursts. Topics that precede demand (Δ*V*/*V* > 0) tend to be about current and possibly unexpected events, such as a military operation in the Middle East and the killing of the U.S. Ambassador in Libya. These articles are created almost instantaneously with the event, to meet the subsequent demand. Articles that follow demand (Δ*V*/*V* < 0) tend to be created in the context of topics that already attract significant attention, such as elections, sport competitions, and anniversaries. For example, the page about Titanic survivor Rhoda Abbott was created in the wake of the 100th anniversary of the sinking.

### Collective attention span

We look at a random subset of 20,000 Wikipedia articles drawn from the previous sample of pages created during the course of 2012. For a generic page, let *t_c_* denote the day it was created. For such page we also define *V_c_* as the volume of traffic in its first week of existence. Analogously, let *V_p_* be the peak daily traffic volume to neighbors measured over an observation window of approximately 60 days centered at *t_c_*, and let *t_p_* be the day of the peak. To detect the peak of traffic to neighbors, we smooth the data with a moving average in a centered 7-day window. To quantify whether a certain amount of visits to a target article constitutes a burst of attention relative to the baseline attention to its topic, in Fig. 3 we plot *V_c_*/*V_p_* as a function of the lag in days *δ* = *t_p_* − *t_c_*, averaged across pages with the same value of *δ*. Error bars represent two standard deviations around the mean. A lag of *δ* = 0 represents a peak of attention that occurs on the day of creation of the new article. Negative/positive values of *δ* indicate that the attention peak occurred *before*/*after* the creation of the article. As shown in the figure, new articles created a few days before or after the peak (−3 ≤ *δ* ≤ +3) tend to receive substantially higher traffic than those created at a later or earlier time. The maximum is achieved when an article is created two or three days after the peak.

## Discussion

### Supply and demand of information

Our result shows that in many cases demand for information precedes its supply. We propose a model to interpret this finding, analogous to the law of supply and demand^26^. An increase in demand indicates a willingness to pay a higher price for a physical good, which in turn leads to an increase in supply. In the domain of information, attention plays the role of price: an increase in demand for information about a topic indicates a higher attention toward that topic, which in turns leads to the generation of additional information about it. This model predicts a causal link between demand and supply of information. Our empirical observations are consistent with this prediction, and may represent a first step toward the development of the fundamental laws of the attention economy.

Whether there is a hard causal link between demand and supply remains an open question. Indeed, other sources of attention, such as conversations on social media, may generate traffic to existing pages as well as trigger the creation of new pages^27^. Our main contribution here has been to establish a quantitative relation between the timing of demand and supply of information. A definition of “information” is more elusive than that of material goods; and quantifying demand is particularly hard in this case.

Another caveat is that not all requests are generated as a result of demand for information. A number of requests to related articles are likely to be generated by the very creators of new entries; one could hardly create new knowledge about some topic without consulting existing pages about it. This is a source of potential bias for our measure of demand especially in the case of low-traffic topics, such as entries about small towns or niche musical bands. On the other hand, significant bursts in volume are observed for popular topics as well (cf. Fig. 2(d)). Such bursts could not possibly be generated by the activity of contributors, who are a small percentage of the Wikipedia audience^28^.

### Collective attention span

In addition to uncovering for the first time a previously unobserved link between collective attention and production of new information, we also find that new articles created shortly before or after the peak of traffic to their related pages tend to garner more views than those created too early or too late. Collective attention concentrates only for a brief period of time — a span of about ±3 days — on these new articles.

### Other signals about content creation

In this study we restricted our notion of content production to the creation of new articles. Wikipedia lets any contributor add further information after the initial publication of an article, but our method does not capture these instances of content creation. As mentioned before, the sequence of revisions to an article is not a good proxy for content production namely because of its noisy nature^18^. To find a signal in it, one would need to identify only “major” updates. Unfortunately, this is hard to do on the basis of text change alone, as the distribution of edit size is heavy-tailed^29^. Furthermore the idiosyncrasies of the editorial process on Wikipedia further complicate things: for example, if a long chunk of text is moved to another article, one would see a large change in terms of text removed and added, even though no novel information has been produced. Devising more sophisticated notions of content creation in this context would be highly desirable in the future.

### Future work

As a practical consequence of our finding, volumetric data about collective attention, such as searches, reviews, and ratings, which now abound online, may be used as indicators of what kinds of new ideas and innovations will ensue.

Our analysis focuses on aggregate-level behaviors. Models of individual browsing behavior could shed more light on how people allocate their attention among competing information stimuli online. Given the sensitive nature of the personal information revealed by individual browsing habits, validating such models with data is a challenge, as revealed by the recent discussions about the trade-offs between data-driven social science research and individual privacy rights. Nevertheless, further empirical analyses and theoretical models of individual and collective dynamics of attention will lead a better understanding of the social exchange of knowledge in online and offline information networks.

## Methods

### Data collection

In our analysis we used the public dataset generated by the servers of the Wikimedia Foundation. Traffic volume is the number of non-unique HTTP requests that an article receives, as a proxy for the popularity of the subject^2^,^13^. We collected data about hourly traffic to the neighbors of Wikipedia articles created during 2012. The data were pre-processed for analysis. We conflated titles that automatically redirect to other entries. We used the information in the ‘redirect’ table to perform this check. We considered only pages created by humans, using a recent list of all known bots to discard automatically-generated pages. Neighbors were found by looking at the ‘pagelinks’ table, after resolving redirects.

### Page creation

To check whether a page was actually created during 2012 we consulted the time stamp of its earliest recorded revision (the reference time stamp). Unfortunately, this information is not always accurate since Wikipedia pages can be merged, migrated, have their edit history fully or partially deleted, or even lost. We thus checked that no traffic to the page had been recorded in our dataset in a 50-week exclusion window before the reference time stamp. However, because it is customary to include links to missing entries in order to encourage other contributors to create them, we found this criterion to produce too many false negatives. We settled for a small threshold, allowing pages with at most 5% (420) non-null hourly observations in the exclusion window.

### Links

At its earliest stage a Wikipedia article rarely contains more than a handful of sentences and links. As a consequence, looking at the early set of neighbors would yield very sparse information. On the other hand, deletion of links is rare^30^. Therefore we collected the neighbors that link to and are linked by the page at the present day.

### Relative traffic change

Let us consider a focal page with *N* neighbours and an observation window of length *L* centered around a reference time *t_c_*, which is the time when the page is created. The total traffic volume each neighbor receives before and after *t_c_* corresponds to random variables 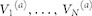 and 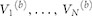, respectively. The average volume change 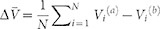 indicates whether, on average, attention to a neighbor is more concentrated before the creation of the page (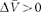) or after (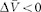).

Even though it accounts for the broad distribution of neighborhood sizes (see Fig. 2(a)), 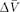 does not guarantee a fair comparison between topics for two reasons: first, the distribution of attention across topics is broad (as shown in Fig. 4); second, Web traffic is known to follow circadian, weekly, and seasonal rhythms^31^. Over a week, an overall change in traffic volume 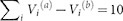 visits may represent a dramatic surge of attention if observed over a group of pages that average 100 visits per week. However, it would be barely noticeable if the same pages averaged 10^4^ visits per week. To overcome these problems, let us define the relative (median) traffic change:
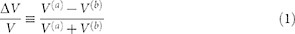
where *V*^(·)^ is the median traffic over a neighbor. We choose to use the median since it is a more robust estimator in the presence of outliers, and almost every article in our samples has at least one very high-traffic neighbor (e.g., “United States”), whose volume of traffic is insensitive to all but the most high-profile events recorded in the dataset. We also repeated our analysis using the sample mean and found qualitatively similar results.

**Figure 1:**
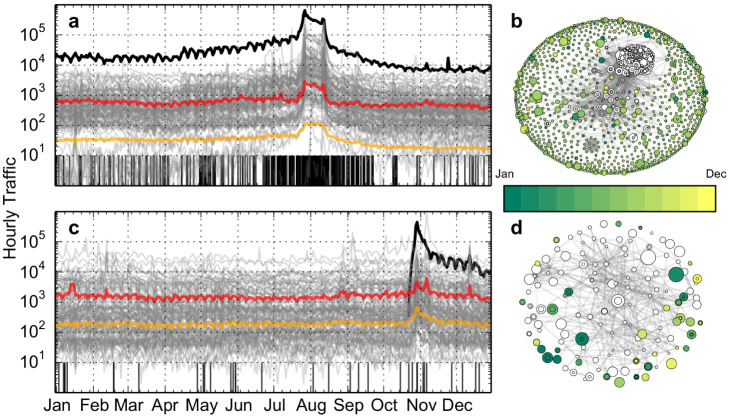
Synchronous traffic bursts associate to increased creation frequency in two high-profile topics. (a), Time series of traffic. The grey lines represent the daily traffic to articles that are linked from/to the article “2012 Summer Olympics,” according to a recent snapshot of Wikipedia (see Methods). For visualization purposes, only a random sample of 100 neighbors is shown. The focal page is represented by the black solid line; red and gold lines represent the average and median traffic, respectively. The vertical black segments represent the times when new linked articles are created (see Methods). (b), Network of neighbors of “2012 Summer Olympics.” White nodes represent the neighbor articles predating 2012; colored nodes correspond to neighbors created in 2012. The size of the nodes is proportional to their yearly traffic volume; their position was computed using the ARF layout^32^. (c and d), Same visualizations as (a) and (b) for the entry about Hurricane Sandy and its neighbors. New articles tend to be peripheral to these networks.

**Figure 2:**
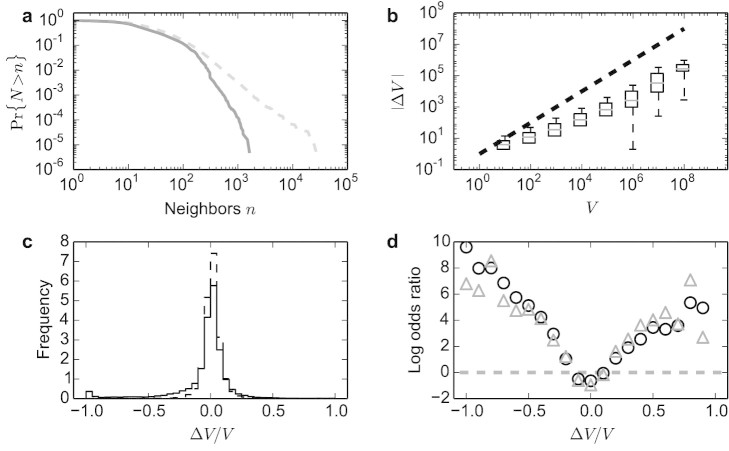
Traffic bursts concomitant with creation of new articles differ from normal traffic patterns. (a), Cumulative distribution of neighborhood size for articles created in 2012 (solid), and pre-existing 2012 (dashed). Neighbors are all articles linking to or from the focal page. Older articles tend to have larger neighborhoods. (b), Absolute infra-week traffic change |Δ*V*| as a function of total traffic volume *V* for articles created in 2012. Even though some topics may receive hundreds of million visits, the change in traffic volume is on average much smaller. Pre-existing pages show a very similar pattern. Boxes stretch from the first to the third quartile, and whiskers represent the 99% confidence interval. Gray segments within boxes indicate the median. The dashed line is a guide to the eye for linear scaling. (c), Distribution of the relative change in traffic volume for 2012 (solid) and pre-existing (dashed) pages. (d), Log odds ratio comparing pages created in 2012 versus existing pages as a function of relative traffic change, for the whole sample (circles), and for a sub-sample of 16,816 pages (18%) with *V* > 2 × 10^5^ visits (triangles). The dashed gray line indicates equal odds.

**Figure 3:**
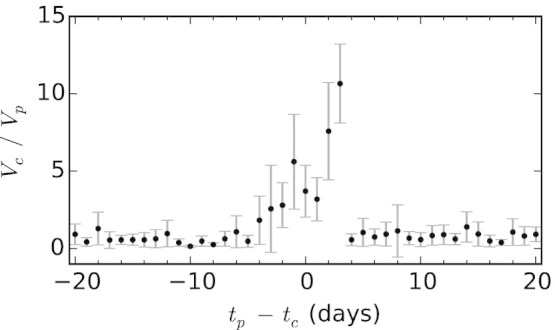
Collective attention span. New articles tend to receive substantially more traffic if created within three days before or after the peak of traffic to their neighboring pages.

**Figure 4:**
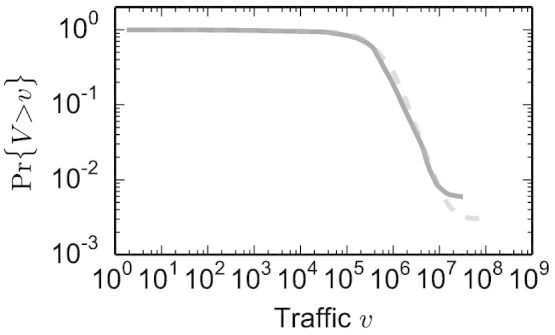
Distribution of traffic volume. Cumulative distribution of total traffic volume *V* for articles created in 2012 (solid) and pre-existing 2012 (dashed).

The length *L* of the observation window must be chosen considering a trade-off between competing requirements. Most attention spikes tend to be relatively brief — on the scale of the day — and so the value of *L* should not be too large, to avoid lumping together consecutive attention bursts. On the other hand, because of the strong circadian and weekly cycles that we see in Fig. 1, *L* cannot be too small, otherwise these fluctuations would dominate the signal for all but the largest bursts. We therefore consider a two-week observation window (*L* = 14 days), centered at the time of creation of the new page.

### Baseline sample

To collect the baseline data we drew at random without replacement an existing page (i.e., created before 2012) for each new page, and extracted traffic to its neighbors at a random time stamp during 2012. We also repeated the analysis with a different baseline sample, where instead of a random time stamp we used the time of creation of the associated new page, and found similar results.

## Author Contributions

G.L.C., A.F. and F.M. designed the research. G.L.C. conducted data collection and analysis. All authors prepared the manuscript.

## Figures and Tables

**Table 1:** Top Wikipedia entries by relative traffic change in absolute value |Δ*V*/*V*|. We consider articles whose neighbors received at least 5 × 10^5^ visits per day, and report the total traffic *V*_tot_ within the entire two-week window

Article	*V*_tot_ (×10^6^ visits)	Δ*V*/*V*
Katherine Copeland	8.66	+0.76
A Symphony of British Music (album)	10.06	+0.69
Elizabeth Price (gymnast)	7.23	+0.65
Operation Pillar of Defense	18.63	+0.49
Lin Qingfeng	8.08	+0.44
J. Christopher Stevens	7.84	+0.44
2013 Australian Open	12.06	+0.43
Niluka Karunaratne	8.75	+0.42
Li Yunqi	8.35	+0.42
Kony 2012	20.42	+0.36
…	…	…
United States presidential election in Idaho, 2012	7.88	−0.22
United States presidential election in Vermont, 2012	7.80	−0.23
United States presidential election in Oklahoma, 2012	7.88	−0.24
United States presidential election in Rhode Island, 2012	7.70	−0.25
United States presidential election in Maryland, 2012	7.02	−0.25
United States presidential election in Illinois, 2012	8.47	−0.26
United States presidential election in Tennessee, 2012	8.06	−0.26
2012 BNP Paribas Open	11.90	−0.34
Rhoda Abbott	7.17	−0.36
Kelley Hurley	8.09	−0.51
